# Supplementary material for: DRP1 induces neuroinflammation via transcriptional regulation of NF-ĸB
Source: Nat Commun. 2026 Mar 16;17:4032. doi: 10.1038/s41467-026-70780-x (PMC13139423; doi:10.1038/s41467-026-70780-x)
Supplement: Supplementary file 1 — Supplementary Information [file 41467_2026_70780_MOESM1_ESM.pdf]

Supplementary Information

DRP1 induces neuroinflammation *via* transcriptional regulation of NF-κB

Yanhao Lai[1,2], Rebecca Z. Fan[1], Harry J. Brown[1,2,3], Said S. Salehe[1], Ethan K. Tieu[1,4], and Kim Tieu[1,2,,5*]

[1]Department of Environmental Health Sciences, Florida International University, Miami, FL, USA.

[2]Biomolecular Sciences Institute, Florida International University, Miami, FL, USA.

[3]College of Arts and Sciences, Florida International University, Miami, FL, USA.

[4]Currrent address: Neuroscience Program, University of Miami, Miami, FL 33136, USA.

[5]Lead contact

*Correspondence: ktieu@fiu.edu (K.T.)

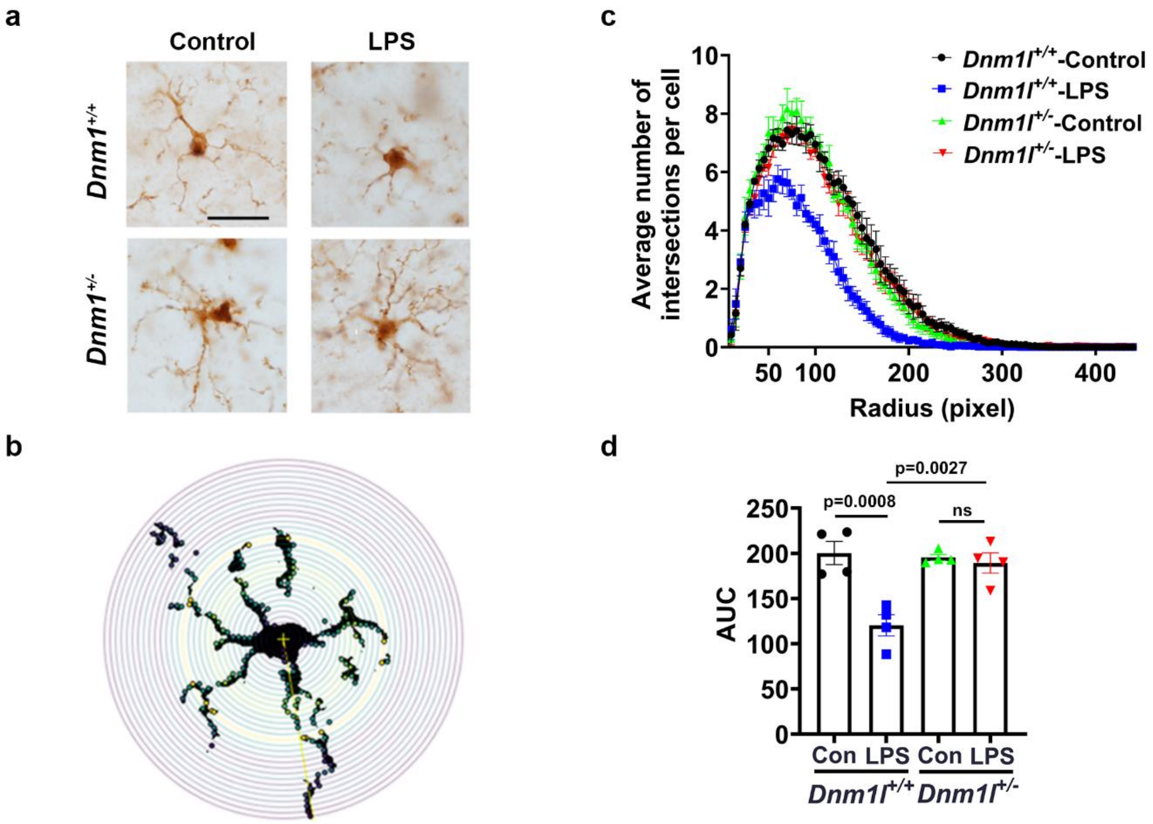

Supplementary Fig. 1 Reduced microglia activation in LPS-treated *Dnm1l*$^{+/-}$ mice.

Three-month-old *Dnm1l*$^{+/-}$ and *Dnm1l*$^{+/+}$ mice received a single intraperitoneal injection of LPS (5 mg/kg) or saline, and ventral midbrains were collected 6 h later for DAB immunostaining (**a**) and Sholl analysis (**b–d**).

a, Coronal midbrain sections were immunostained for IBA1 and visualized using DAB. For each animal, images of 30 microglia from the substantia nigra were captured at 100× magnification (*N* = 4 mice; 2M & 2F). Scale bar = 50 μm.

b, Morphological changes in microglia were quantified using Sholl analysis, performed by investigators blinded to treatment groups.

c, Average number of intersections per cell derived from Sholl analysis.

d, Area under the curve (AUC) generated from Sholl profiles and quantified. Data are presented as mean ± SEM; two-way ANOVA followed by Tukey's post hoc test.

Source data are provided as a Source Data file.

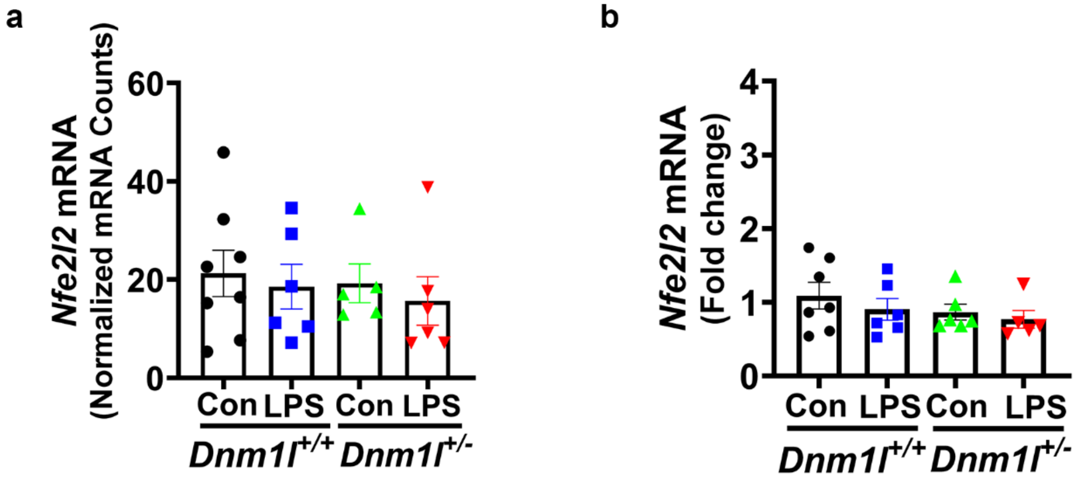

Supplementary Fig. 2 *Nfe2l2* levels in LPS-treated mice.

Three-month-old *Dnm1l*$^{+/-}$ and *Dnm1l*$^{+/+}$ mice received a single intraperitoneal injection of LPS (5 mg/kg) or saline, and ventral midbrains were collected 6 h later for NanoString nCounter gene expression analysis **(a)** and qPCR validation **(b)**.

a, *Nfe2l2* mRNA levels were quantified by NanoString and normalized to the reference genes *Aars*, *Ccdc127*, *Cnot10*, *Tada2b*, and *Xpnpep1*. *N* = 5–8 mice per group (2–4F & 3–4M); Data are presented as mean ± SEM, two-way ANOVA.

b, qPCR analysis of *Nfe2l2* expression in the ventral midbrains (normalized to *Gapdh*). *N* = 5–7 mice per group (2–4F & 3–4M); Data are presented as mean ± SEM, two-way ANOVA followed by Tukey's post hoc test.

Source data are provided as a Source Data file.

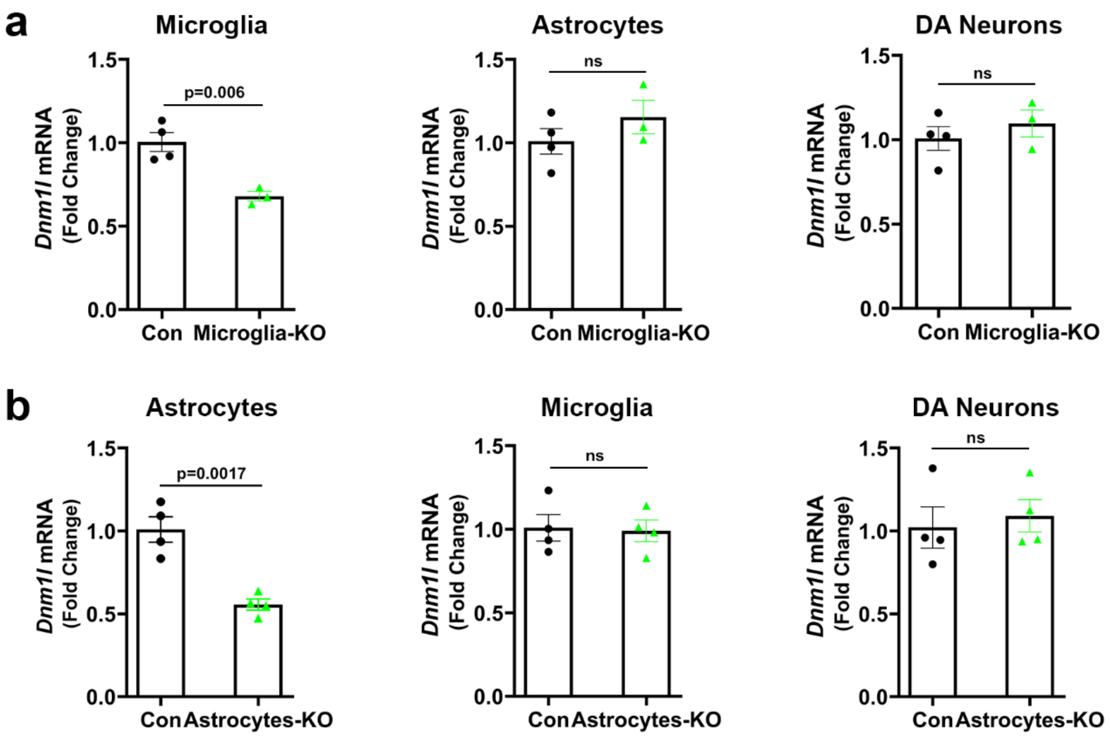

Supplementary Fig. 3 *Dnm1l* expression in different cell types from conditional *Dnm1l*-KO mouse models.

Brains were collected from 3-month-old “Microglia-KO” (**a**) and “Astrocyte-KO” (**b**) mice three weeks after tamoxifen injection. Tamoxifen-treated *Dnm1l-Loxp*$^{+/-}$ mice served as controls. Snap-frozen brains were sectioned at 10 µm onto PEN membrane slides and immunostained with antibodies against IBA1, GFAP, and TH to identify microglia, astrocytes, and DA neurons, respectively. Individual cells were isolated by laser microdissection. Using the Smart-seq2 method, cDNA from 40 cells of each cell type per animal was pre-amplified and analyzed by qPCR to quantify *Dnm1l* expression (normalized to *Gapdh*). *N* = 3–4 mice per group (1–2F & 2M). Data represent mean ± SEM; unpaired two-sided t-tests. Source data are provided as a Source Data file.

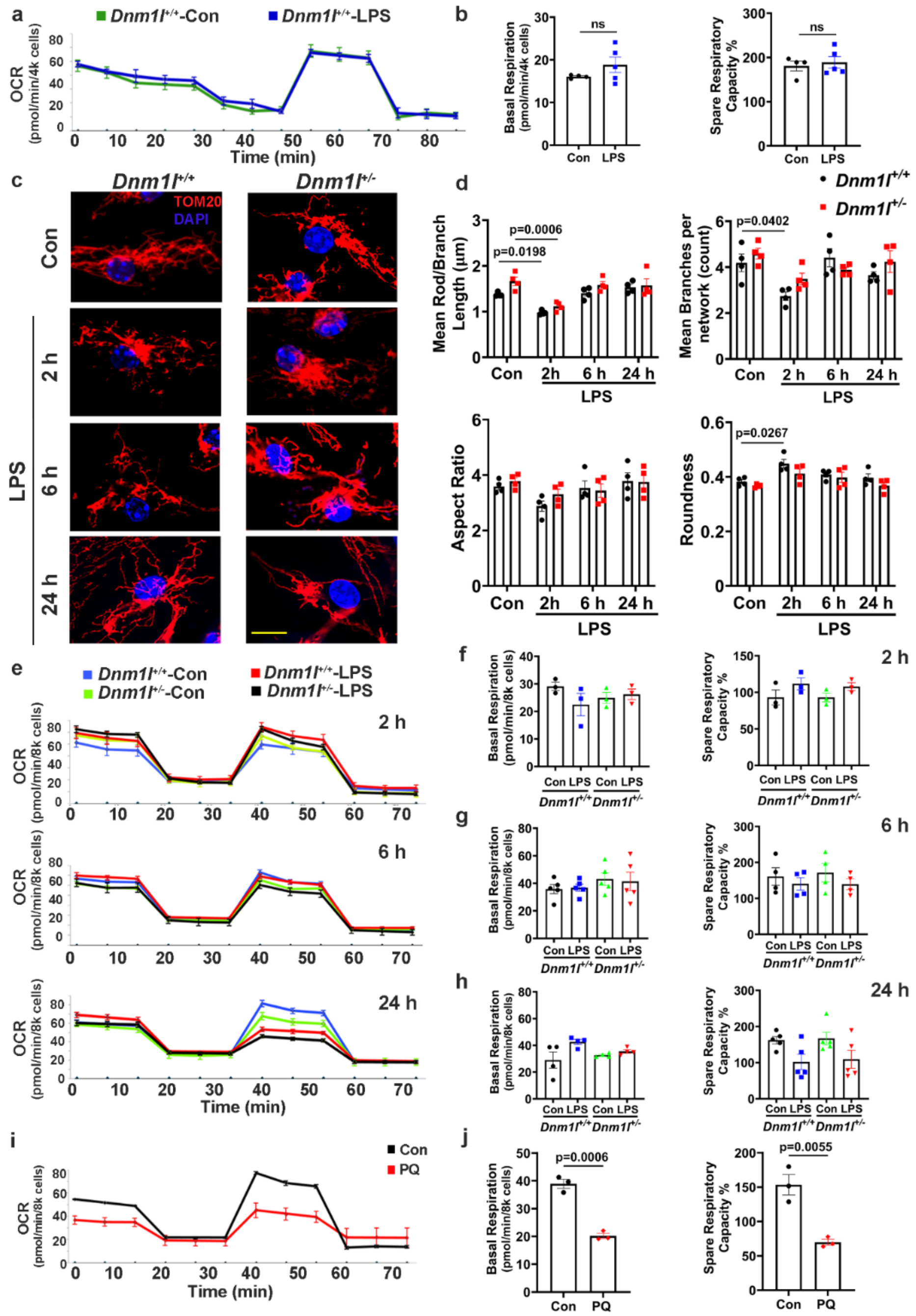

Supplementary Fig. 4 Mitochondrial morphology and function in LPS-treated microglia. **a–b**, Adult *Dnm1l*$^{+/+}$ mice (2–4 months old) were injected with LPS (5 mg/kg, i.p.) or saline for 6 h. Brain microglia were isolated and enriched for mitochondrial respiration analysis. **a**, Representative kinetic traces from the mito-stress assay. **b**, Quantified respiration parameters. Data represent mean ± SEM; *N* = 4–5 mice (2F & 2–3M) per group; unpaired two-sided t-tests.

c–d, Primary microglia derived from *Dnm1l*$^{+/+}$ and *Dnm1l*$^{+/-}$ pups were treated with LPS (100 ng/mL) or vehicle for 2 h, 6 h, or 24 h. **c**, Representative TOM20 immunofluorescence images showing mitochondrial morphology. Scale bar = 10 μm. **d**, Mitochondrial network morphology quantified using the Mitochondrial Network Analysis (MiNA) Fiji plugin. *N* = 4 independent experiments, 20 cells/experiment.

e–j, Mitochondrial respiration of primary microglia was measured using the XFe96 Extracellular Flux Analyzer. **e**, Representative mito-stress assay traces at 2 h, 6 h, and 24 h post-LPS treatment. **f–h**, Quantification of oxygen consumption rate (OCR) at 2 h (f), 6 h (g), and 24 h (h). *N* = 3 independent experiments (2 h) or 4–5 experiments (6 h, 24 h), each with 6–8 technical replicates; Data represent mean ± SEM, two-way ANOVA with Tukey's post hoc test. **i–j**, Positive control for mitochondrial dysfunction. Microglia were treated with 100 μM paraquat (PQ) for 24 h prior to mito-stress testing. **i**, Representative kinetic traces. **j**, Quantified OCR. *N* = 3 independent experiments with 6–8 technical replicates; unpaired two-sided t-tests. Source data are provided as a Source Data file.

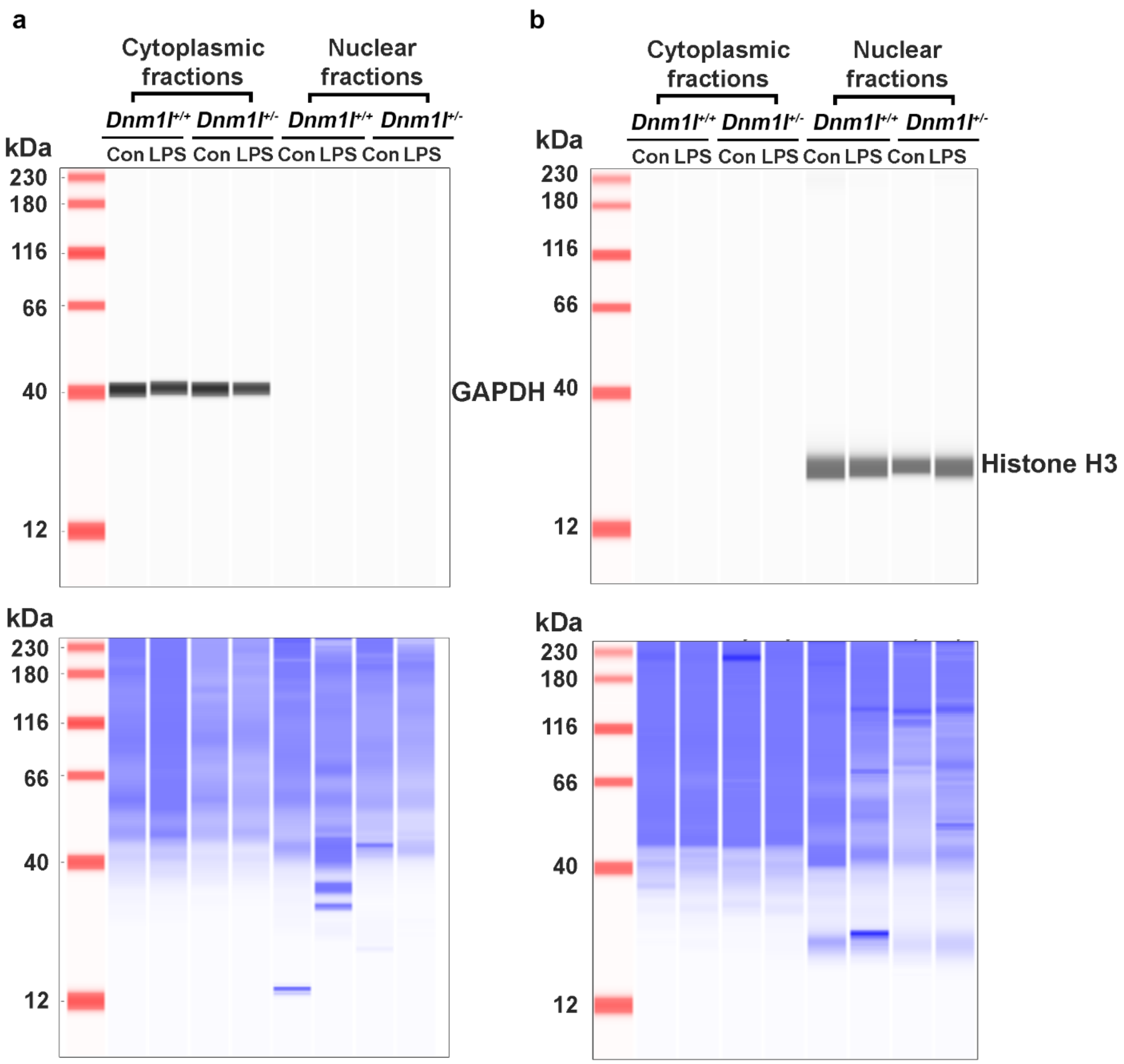

Supplementary Fig. 5 Purity of cytoplasmic and nuclear fractions.

Primary microglia from *Dnm1l*$^{+/+}$ and *Dnm1l*$^{+/-}$ pups were treated with LPS (100 ng/mL, 6 h) and subjected to subcellular fractionation. **a,** Immunoblot analysis of GAPDH, a cytoplasmic marker, in cytoplasmic and nuclear fractions. **b,** Immunoblot analysis of Histone H3, a nuclear marker, in the same fractions. Total protein staining (bottom panels) was used as a loading control. The absence of GAPDH in nuclear fractions and the absence of Histone H3 in cytoplasmic fractions confirm the purity of the separated compartments. Source data are provided as a Source Data file.

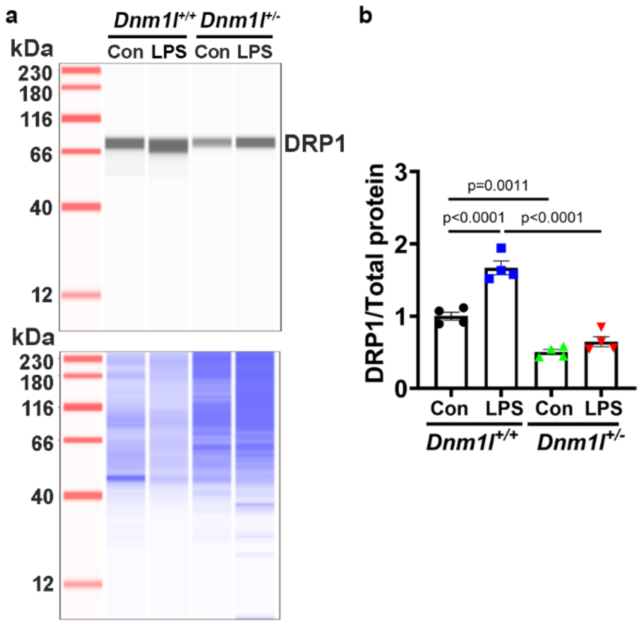

Supplementary Fig. 6. DRP1 deficiency prevents LPS-induced upregulation of DRP1 in whole-cell lysates.

Primary microglia from *Dnm1l*$^{+/+}$ and *Dnm1l*$^{+/-}$ pups were treated with LPS (100 ng/mL, 6 h), followed by preparation of whole-cell lysates using RIPA buffer. **a**, Immunoblot analysis of DRP1 in whole-cell lysates (top panel). Total protein staining (bottom panel) served as loading control. **b**, Quantification of DRP1 protein levels shown in **(a)**. Data represent mean ± SEM from $N = 4$ independent experiments; two-way ANOVA with Tukey's post hoc test. Source data are provided as a Source Data file.

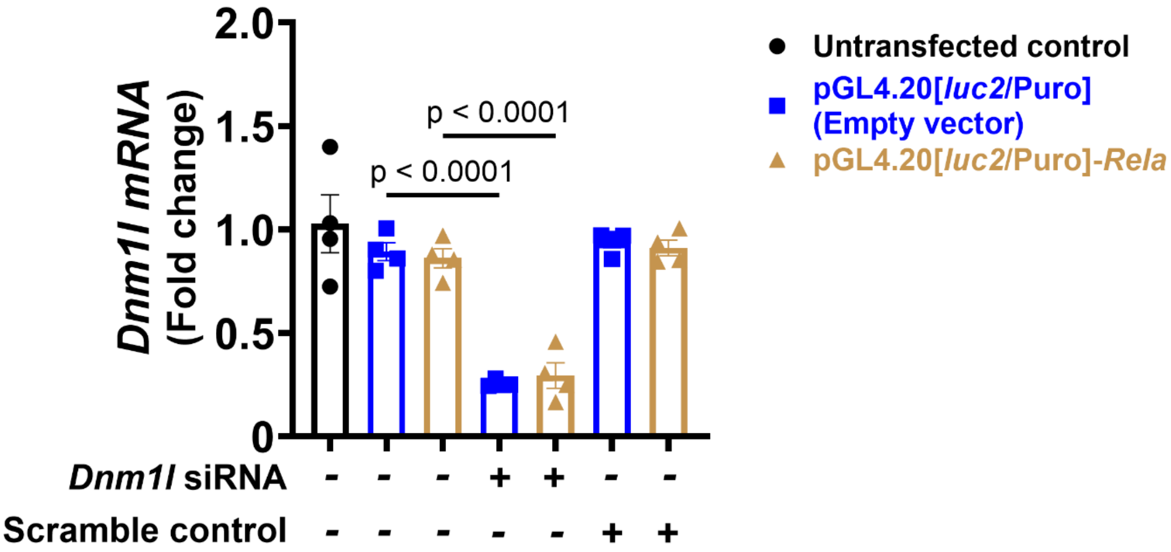

Supplementary Fig. 7. Confirmation of *Dnm1l* siRNA–mediated gene knockdown in HT22 cells.

HT22 cells were transfected for 24 h with either pGL4.20[*luc2*/Puro] or pGL4.20[*luc2*/Puro]-*Rela* in the presence or absence of *Dnm1l* siRNA or scrambled control siRNA. Untransfected cells served as an additional control. Forty-eight hours after transfection, cells were harvested for qPCR analysis of *Dnm1l* expression (normalized to *Gapdh*). Data represent mean ± SEM from $N = 4$ independent experiments; one-way ANOVA followed by Tukey's post hoc test. Source data are provided as a Source Data file.

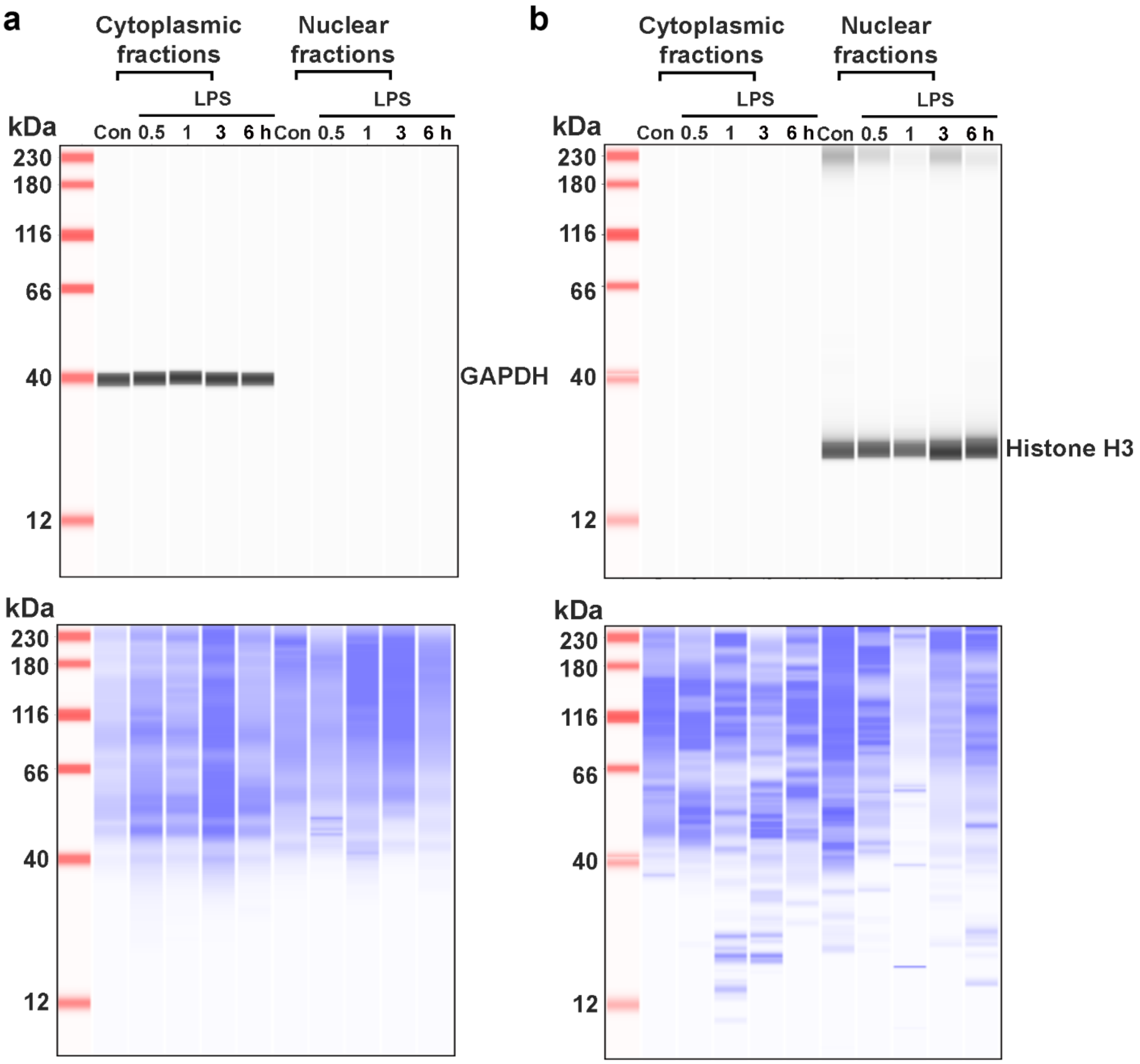

Supplementary Fig. 8 Purity of cytoplasmic and nuclear fractions in primary microglia

WT (*Dnm1l*$^{+/+}$) primary microglia were treated with vehicle or 100 ng/mL LPS for 0.5, 1, 3, or 6 h. Fractionated cytoplasmic and nuclear extracts were assessed by immunoblotting for **(a)** GAPDH (cytoplasmic marker) and **(b)** Histone H3 (nuclear marker) to evaluate fraction purity (top panels). Total protein staining (bottom panels) was used as the loading control. Source data are provided as a Source Data file.

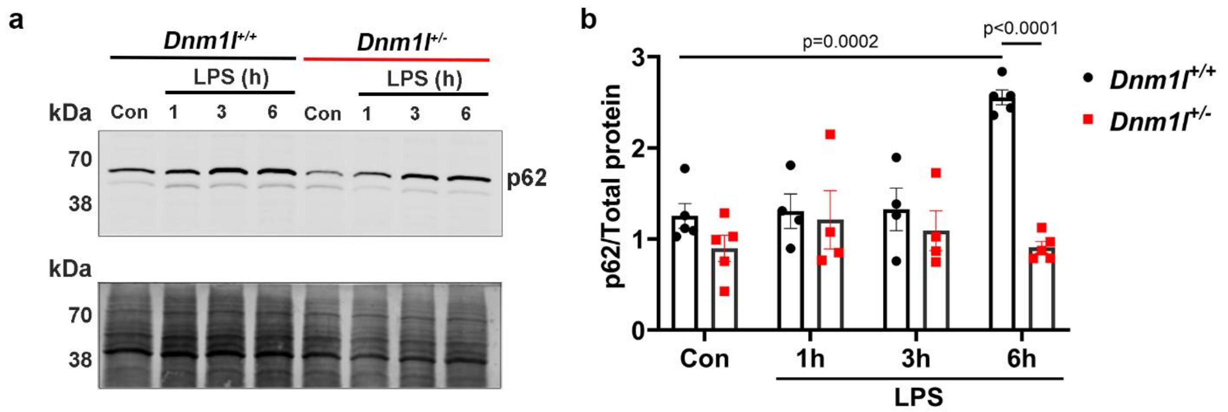

Supplementary Fig. 9 DRP1-KO protects against LPS-induced impairment of autophagy. Primary *Dnm1l*$^{+/+}$ and *Dnm1l*$^{+/-}$ microglia were treated with 100 ng/mL LPS for 1, 3, or 6 h; vehicle-treated cells served as controls. **a**, SDS-PAGE immunoblot analysis of p62 in whole-cell lysates. Total protein staining (bottom panels) was used as the loading control. **b**, Quantification of p62 levels shown in **(a)**. Data represent mean ± SEM from *N* = 4–5 independent experiments; two-way ANOVA followed by Tukey's post hoc test. Source data are provided as a Source Data file.

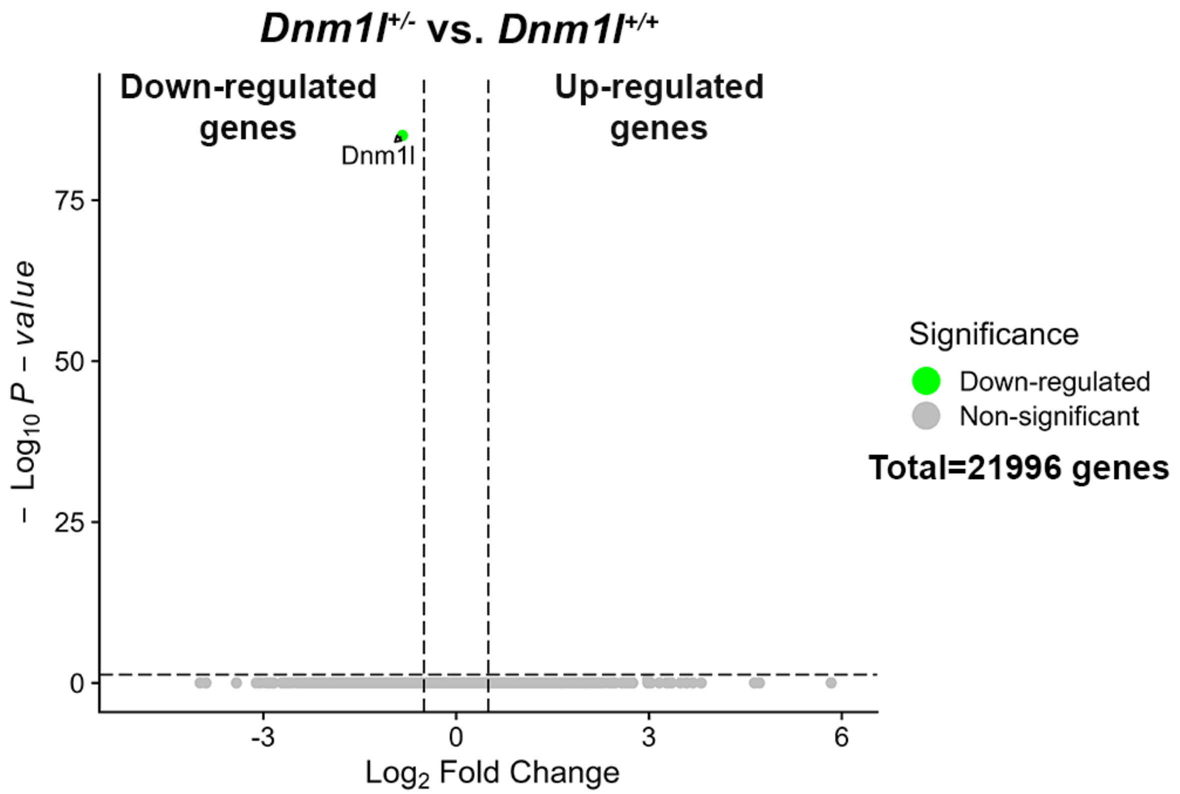

Supplementary Fig. 10 Differentially expressed genes (DEGs) identified by RNA-seq in *Dnm1l*+/- mice compared with *Dnm1l*$^{+/+}$ littermates.

Ventral midbrains from 3-month-old *Dnm1l*$^{+/-}$ and *Dnm1l*$^{+/+}$ mice were collected for RNA extraction using TRIzol and subsequent RNA-seq analysis. The volcano plot illustrates DEGs between genotypes (threshold: 0.05-fold change, $p < 0.05$). $N = 8$ mice per group (4F, 4M). Source data are provided as a Source Data file.

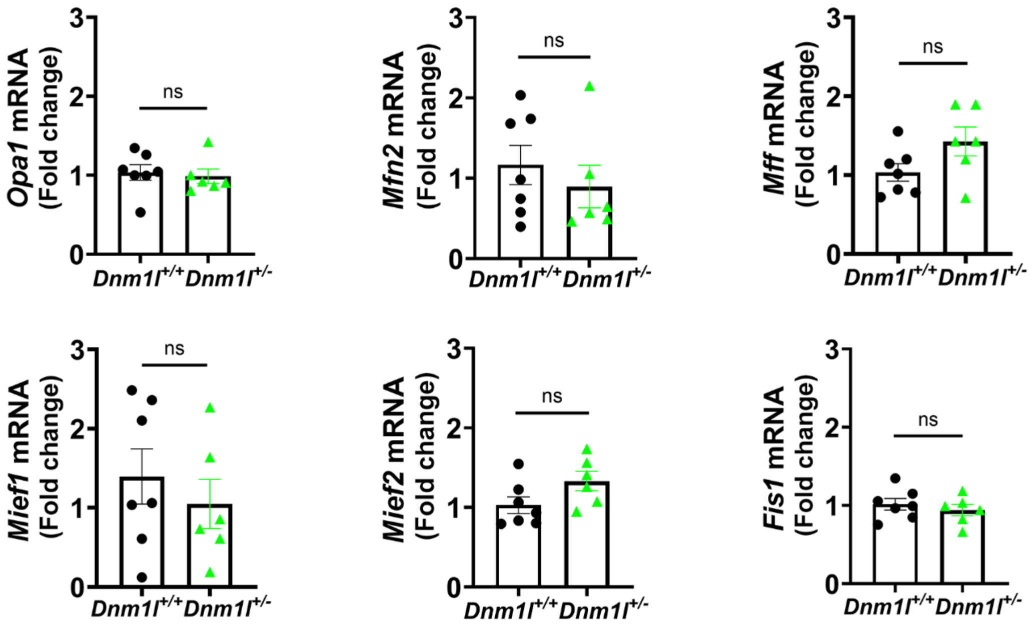

Supplementary Fig. 11 Mitochondrial dynamics regulators in *Dnm1l*$^{+/-}$ mice compared with *Dnm1l*$^{+/+}$ littermates.

Ventral midbrains from 3-month-old *Dnm1l*$^{+/-}$ and *Dnm1l*$^{+/+}$ mice were collected for RNA extraction and qPCR analysis. Relative expression levels of mitochondrial dynamic-related genes (*Opa1*, *Mfn2*, *Mff*, *Mief1*, *Mief2*, and *Fis1*) were quantified using *Gapdh* as the reference gene. *N* = 6–7 mice per group (2–3F and 4M); Data are presented as mean ± SEM, unpaired two-sided t-tests. Source data are provided as a Source Data file.

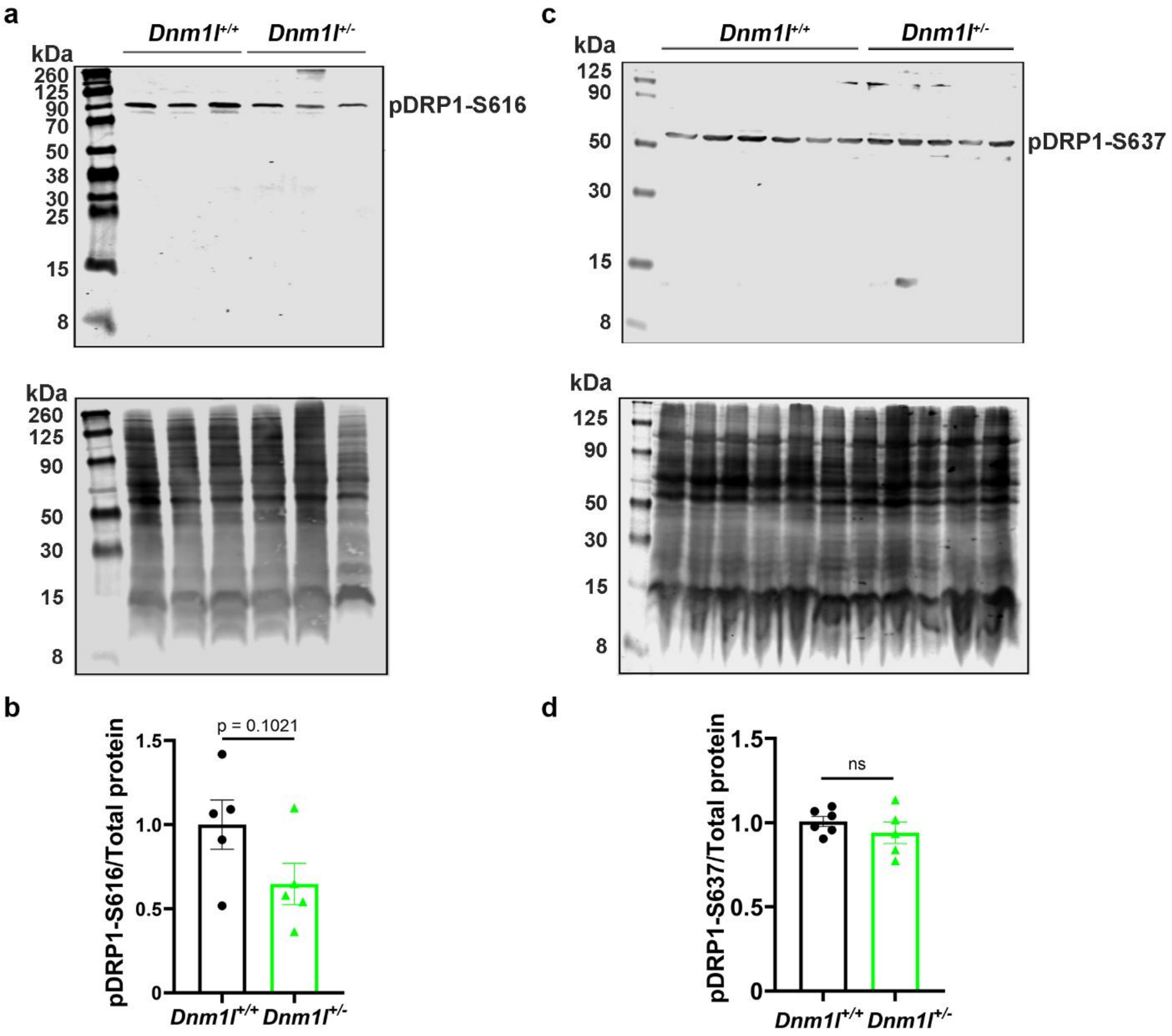

Supplementary Fig. 12. Protein levels of pDRP1-S616 and pDRP1-S637 in *Dnm1l*$^{+/+}$ and *Dnm1l*$^{+/-}$ mice.

Microdissected ventral midbrains from *Dnm1l*$^{+/+}$ and *Dnm1l*$^{+/-}$ mice was processed for SDS-PAGE immunoblotting to assess phosphorylation of DRP1 at Ser616 **(a)** and Ser637 **(c)**. Interesting, pDRP1-S637 migrates faster than pDRP1-S616 in mouse brain lysates. Total protein staining (bottom panels) was used as the loading control. **b**, Quantification of pDRP1-S616 levels from **(a)**. **d**, Quantification of pDRP1-S637 levels from **(c)**. *N* = 5–6 mice per group (2–4F and 2–4M); Data are presented as mean ± SEM, unpaired two-sided *t*-tests. Source data are provided as a Source Data file.

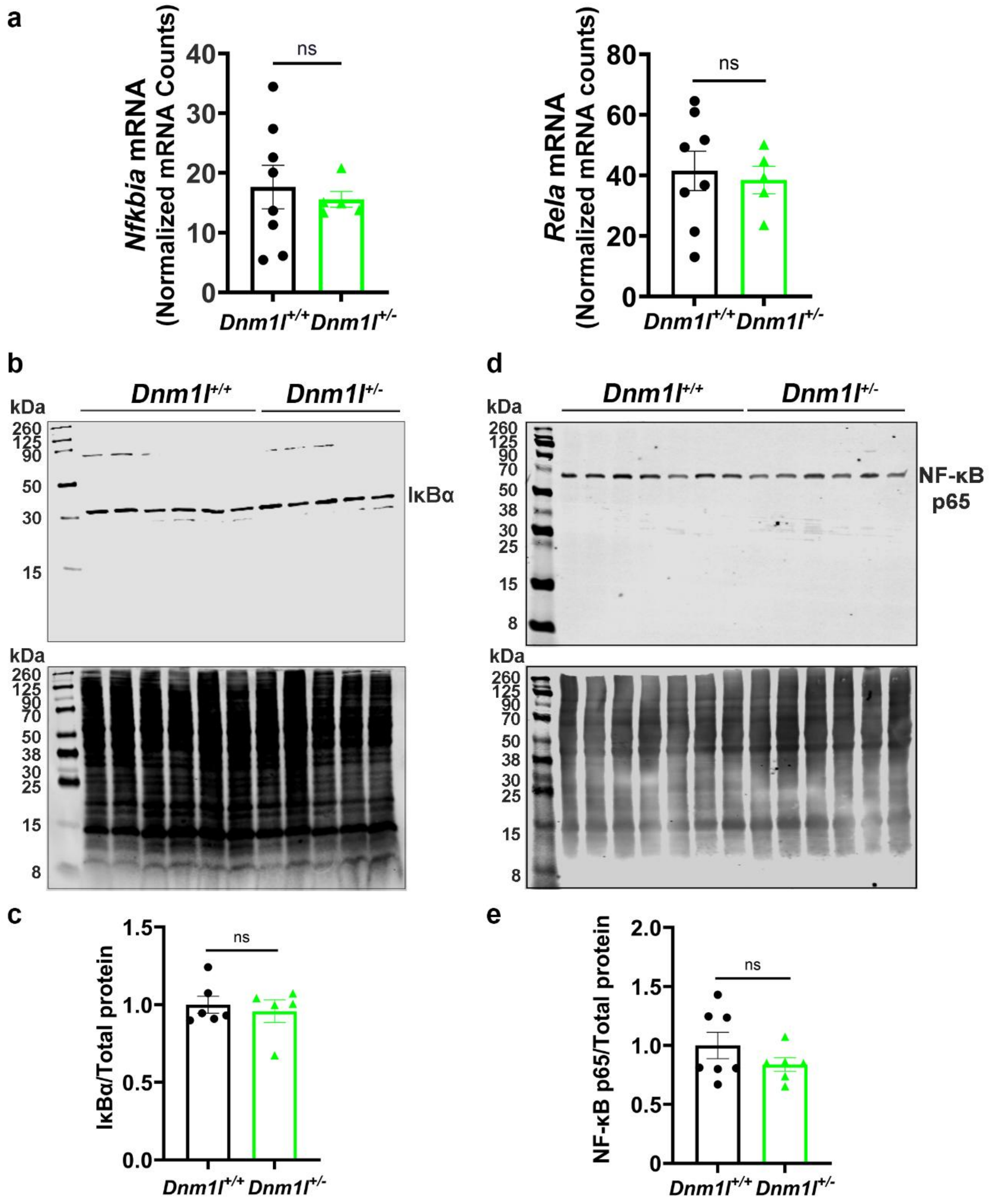

Supplementary Fig. 13 mRNA and protein levels of *Nfkbia*/IκBα and *Rela*/p65 in *Dnm1l*$^{+/-}$ mice.

a, NanoString nCounter gene expression analysis of *Nfkbia* and *Rela* in the ventral midbrains of *Dnm1l*$^{+/+}$ and *Dnm1l*$^{+/-}$ mice. **b–c**, Immunoblotting of IκBα and corresponding quantification. **d–e**, Immunoblotting of NFκB-p65 and corresponding quantification. *N* = 6–7 mice per group (2–3F and 3–4M); Data are presented as mean ± SEM, unpaired two-sided t-tests. Source data are provided as a Source Data file.
