# Supplementary material for: DRP1 induces neuroinflammation via transcriptional regulation of NF-ĸB
Source: Nat Commun. 2026 Mar 16;17:4032. doi: 10.1038/s41467-026-70780-x (PMC13139423; doi:10.1038/s41467-026-70780-x)
Supplement: Supplementary file 2 — Description of Additional Supplementary Files [file 41467_2026_70780_MOESM2_ESM.pdf]

Description of Additional Supplementary Files

File name: Supplementary Data 1

Description: NanoString ratio data.

File name: Supplementary Data 2

Description: Oligonucleotides used for ChIP-qPCR.

File name: Supplementary Data 3

Description: F statistics for ANOVA.
