# Supplementary material for: DRP1 induces neuroinflammation via transcriptional regulation of NF-ĸB
Source: Nat Commun. 2026 Mar 16;17:4032. doi: 10.1038/s41467-026-70780-x (PMC13139423; doi:10.1038/s41467-026-70780-x)
Supplement: Supplementary file 6 — Reporting Summary [file 41467_2026_70780_MOESM6_ESM.pdf]

nature portfolio

Corresponding author(s): Kim Tieu

Last updated by author(s): Feb 8, 2026

Reporting Summary

Nature Portfolio wishes to improve the reproducibility of the work that we publish. This form provides structure for consistency and transparency in reporting. For further information on Nature Portfolio policies, see our Editorial Policies and the Editorial Policy Checklist.

Statistics

For all statistical analyses, confirm that the following items are present in the figure legend, table legend, main text, or Methods section.

n/a	Confirmed	
☐	☒	The exact sample size (*n*) for each experimental group/condition, given as a discrete number and unit of measurement
☐	☒	A statement on whether measurements were taken from distinct samples or whether the same sample was measured repeatedly
☐	☒	The statistical test(s) used AND whether they are one- or two-sided<br>*Only common tests should be described solely by name; describe more complex techniques in the Methods section.*
☐	☒	A description of all covariates tested
☐	☒	A description of any assumptions or corrections, such as tests of normality and adjustment for multiple comparisons
☐	☒	A full description of the statistical parameters including central tendency (e.g. means) or other basic estimates (e.g. regression coefficient) AND variation (e.g. standard deviation) or associated estimates of uncertainty (e.g. confidence intervals)
☐	☒	For null hypothesis testing, the test statistic (e.g. *F*, *t*, *r*) with confidence intervals, effect sizes, degrees of freedom and *P* value noted<br>*Give P values as exact values whenever suitable.*
☒	☐	For Bayesian analysis, information on the choice of priors and Markov chain Monte Carlo settings
☒	☐	For hierarchical and complex designs, identification of the appropriate level for tests and full reporting of outcomes
☒	☐	Estimates of effect sizes (e.g. Cohen's *d*, Pearson's *r*), indicating how they were calculated

Our web collection on statistics for biologists contains articles on many of the points above.

Software and code

Policy information about availability of computer code

Data collection	nSolver v4.0 software<br>QuantStudio Software v1.7.2<br>MSD QuickPlex SQ 120 and Discovery Workbench software<br>Sholl Analysis plug-in for Fiji<br>Mitochondrial Network Analysis (MiNA) plugin in Fiji<br>Imaris 10.0<br>Compass software v6.0.<br>SoftMax Pro 7.0.3
Data analysis	All statistical analysis were performed using GraphPad Prism version 10.6.1. Volcano plots were generated using the Positron integrated development environment (version 2025.11.0, build 234; Posit PBC) with Bioconductor version 3.22 and the EnhancedVolcano package version 1.27.

For manuscripts utilizing custom algorithms or software that are central to the research but not yet described in published literature, software must be made available to editors and reviewers. We strongly encourage code deposition in a community repository (e.g. GitHub). See the Nature Portfolio guidelines for submitting code & software for further information.

Data

Policy information about availability of data

All manuscripts must include a data availability statement. This statement should provide the following information, where applicable:

- Accession codes, unique identifiers, or web links for publicly available datasets
- A description of any restrictions on data availability
- For clinical datasets or third party data, please ensure that the statement adheres to our policy

All data supporting the findings of this study are available within the article as well as Supplementary Information and Source Data files. Source data are provided with this paper.

Research involving human participants, their data, or biological material

Policy information about studies with human participants or human data. See also policy information about sex, gender (identity/presentation), and sexual orientation and race, ethnicity and racism.

Reporting on sex and gender	N/A
Reporting on race, ethnicity, or other socially relevant groupings	N/A
Population characteristics	N/A
Recruitment	N/A
Ethics oversight	N/A

Note that full information on the approval of the study protocol must also be provided in the manuscript.

Field-specific reporting

Please select the one below that is the best fit for your research. If you are not sure, read the appropriate sections before making your selection.

☒ Life sciences ☐ Behavioural & social sciences ☐ Ecological, evolutionary & environmental sciences

For a reference copy of the document with all sections, see nature.com/documents/nr-reporting-summary-flat.pdf

Life sciences study design

All studies must disclose on these points even when the disclosure is negative.

Sample size	For animal experiments, N=4-10 animals per group as indicated in the Figure Legends. Sample size was determined by an a priori power analysis (α = 0.05, two-sided; power = 0.80) based on the expected effect size and variability from our preliminary data.
Data exclusions	Data were excluded for data analysis if they failed passing the ROUT outlier tests.
Replication	For cell experiments, N=3-4 independent experiments as indicated in the Figure Legends.
Randomization	All animals and/or cells used in this study were randomized into treatment groups.
Blinding	Investigators were blinded to group allocation during data collection and/or data analysis.

Reporting for specific materials, systems and methods

We require information from authors about some types of materials, experimental systems and methods used in many studies. Here, indicate whether each material, system or method listed is relevant to your study. If you are not sure if a list item applies to your research, read the appropriate section before selecting a response.

Materials & experimental systems

n/a	Involved in the study
☐	☒ Antibodies
☐	☒ Eukaryotic cell lines
☒	☐ Palaeontology and archaeology
☐	☒ Animals and other organisms
☒	☐ Clinical data
☒	☐ Dual use research of concern
☒	☐ Plants

Methods

n/a	Involved in the study
☒	☐ ChIP-seq
☒	☐ Flow cytometry
☒	☐ MRI-based neuroimaging

Antibodies

Antibodies used

Rabbit anti-IBA1, cat. no. 019-19741, FUJIFILM Wako Chemicals;
Chicken anti-tyrosine hydroxylase, cat. no. ab76442, Abcam;
Mouse anti-GAD67, cat. no. MAB5406, Millipore;
Chicken anti-GFAP, cat. no. PA1-10004, Thermo Fisher;
Rabbit anti-TOM20, cat. no. ab186735, Abcam;
Mouse anti-DRP1, cat. no. 611113, BD Biosciences;
Rabbit anti-DRP1, cat. no. NB110-55288, Novus Biologicals;
Goat anti-IBA1, cat. no. NB100-1028, Novus Biologicals;
Rabbit anti–NF-κB p65, cat. no. 8242, Cell Signaling Technology (CST);
Rabbit anti-Histone H3, cat. no. ab1791, Abcam;
Rabbit anti-GAPDH, cat. no. 2118, CST;
Rabbit anti–pDRP1-Ser616, cat. no. 4494, CST;
Rabbit anti–pDRP1-Ser637, cat. no. GTX01567, GeneTex;
Mouse anti-IκBα, cat. no. NB100-56507, Novus Biologicals;
Rabbit anti-p62, cat. no. PM045, MBL;
Rabbit IgG control, cat. no. 2729, CST;
Rabbit anti-DRP1, cat. no. 8570, CST;
Biotinylated goat anti-rabbit IgG, cat. no. BA-1100, Vector Laboratories;
Alexa Fluor 568 goat anti-chicken, cat. no. A11041, Thermo Fisher;
Alexa Fluor 488 goat anti-rabbit, cat. no. A11008, Thermo Fisher;
Alexa Fluor 488 goat anti-mouse, cat. no. A11029, Thermo Fisher;
Alexa Fluor 488 donkey anti-rabbit, cat. no. A32790, Thermo Fisher;
Alexa Fluor 633 donkey anti-goat, cat. no. A21082, Thermo Fisher;
Anti-rabbit IgG (H+L) DyLight® 800, cat. no. 5151, CST;
Anti-mouse IgG (H+L) DyLight® 800, cat. no. 5257, CST.

Validation

All the antibodies used were validated by the manufacturer as indicated.

Eukaryotic cell lines

Policy information about cell lines and Sex and Gender in Research

Cell line source(s)	HT-22 Mouse Hippocampal Neuronal Cell Line (CAT#SCC129, Sigma-Aldrich)
Authentication	This is an immortalized cell line which is commercial available through Sigma-Aldrich. Thus, the authentication was fulfilled by Sigma-Aldrich as well.
Mycoplasma contamination	Cells are negative for Mycoplasma contamination.
Commonly misidentified lines (See ICLAC register)	N/A

Animals and other research organisms

Policy information about studies involving animals; ARRIVE guidelines recommended for reporting animal research, and Sex and Gender in Research

Laboratory animals	For all animals used in this study, strains and age were specified in the "Methods" and corresponding Figure Legends.
Wild animals	This study did not involve wild animals.
Reporting on sex	Male and female mice were randomized into treatment groups. No sex differences were detectable, and data were combined.

Field-collected samples	This study did not involve samples collected from the field.
Ethics oversight	All mice used in this study were bred, maintained, and characterized in the animal care facility at Florida International University (FIU). All procedures were approved by, and conducted in accordance with, the FIU Institutional Animal Care and Use Committee (Protocol Approval #: IACUC-24-088-CR01).

Note that full information on the approval of the study protocol must also be provided in the manuscript.

Plants

Seed stocks	N/A
Novel plant genotypes	N/A
Authentication	N/A
